# Comprehensive Bioinformatics Analysis to Reveal Key RNA Targets and Hub Competitive Endogenous RNA Network of Keratoconus

**DOI:** 10.3389/fgene.2022.896780

**Published:** 2022-06-07

**Authors:** Shuai Ouyang, Jingyu Ma, Qihang Sun, Jinyan Li, Yijia Chen, Lixia Luo

**Affiliations:** State Key Laboratory of Ophthalmology, Guangdong Provincial Key Laboratory of Ophthalmology and Visual Science, Zhongshan Ophthalmic Centre, Sun Yat-sen University, Guangzhou, China

**Keywords:** Keratoconus, competitive endogenous RNA network, bioinformatics analysis, miRNA, ubiquitin

## Abstract

Keratoconus (KC) is the most common corneal ectatic disease, with its pathological mechanisms unclear. We mainly performed bioinformatics approaches to reveal core RNA targets and hub competitive endogenous RNA (ceRNA) network and explored the potential regulatory mechanisms of ceRNA in KC. The high-throughput sequencing datasets GSE77938 and GSE151631 were downloaded from the Gene Expression Omnibus (GEO) database. The differential expression of mRNAs and lncRNAs was identified using the DESeq2 package. Functional enrichment analyses and protein–protein interaction (PPI) were executed. Then, the hub genes were filtered and molecular docking analysis was performed. Moreover, we predicted miRNAs through a website database and validated them using quantitative PCR (qPCR). Eventually, the lncRNA–miRNA–mRNA regulatory network was constructed by Cytoscape. We revealed that 428 intersected differentially expressed mRNA (DEGs) and 68 intersected differentially expressed lncRNA (DELs) were shared between the two datasets. Functional enrichment results innovatively showed that the ubiquitin-dependent protein catabolic process was upregulated in KC. The pathway enrichment showed that DEGs were mainly involved in NF-kB signaling and neurodegenerative diseases. In addition, we uncovered the top 20 hub genes in which *FBXW11*, *FBXO9*, *RCHY1*, and *CD36* were validated by qPCR. Particularly, a small-molecule drug triptolide was predicted by molecular docking to be a candidate drug for treating KC. Moreover, we innovatively predicted and validated four core miRNAs (miR-4257, miR-4494, miR-4263, and miR-4298) and constructed a ceRNA network that contained 165 mRNA, eight lncRNAs, and four core miRNAs. Finally, we proposed a potential regulatory mechanism for KC. Overall, we uncovered a hub ceRNA network that might underlie a critical posttranslational regulatory mechanism in KC, in which miR-4257, miR-4494, miR-4263, and miR-4298 could be valuable biomarkers and provided core RNAs therapeutic targets for KC.

## Introduction

Keratoconus (KC) is the most common corneal ectatic disease which is characterized by a swollen and thinned cone-shaped cornea, and most patients need corneal transplantation in severe stages. It has been reported that genetic and environmental factors may contribute to this disease (Lucas and Burdon, 2020; McComish et al., 2020). High-throughput transcriptome sequencing in recent years has revealed a part of altered transcript levels in KC, such as disorder of collagen synthesis and catabolic pathway, and the expression of antioxidant genes regulated by NRF2 was significantly decreased (Kabza et al., 2017; Shinde et al., 2020). These findings suggest that abnormalities in oxidative stress levels and collagen degradation levels occur in KC. However, its pathological mechanisms are still unclear, and non-surgical therapy such as drug therapy is still lacking.

MicroRNA (miRNA) is a small single-stranded non-coding RNA (ncRNA) molecule that inhibits translation or increases the degradation of target messenger RNAs (mRNAs) to downregulate gene expression at the posttranscriptional level (Fabian et al., 2010). Moreover, miRNAs have the potential to serve as disease biomarkers (van den Berg et al., 2020). Altered miRNA expression levels in corneal epithelial cells of KC had been reported and 12 miRNAs were downregulated in KC which is involved in cell junction and motor activity (Wang Yu et al., 2018). Long non-coding RNAs (lncRNA) are a type of RNA longer than 200 nucleotides in length and not translated into protein (Kung et al., 2013). According to the competitive endogenous RNA (ceRNA) theory, lncRNAs can compete with mRNAs for binding to miRNAs, which in turn modulates gene expression (Salmena et al., 2011). A large number of lncRNAs were identified to be related to KC, which are involved in cytokine response and cell adhesion (Khaled et al., 2018); however, the research on core lncRNA–miRNA–mRNA regulatory networks and specific key RNA targets of KC has remained largely unexplored.

In this study, the differentially expressed (DE)-RNAs between KC samples and control samples were screened by analyzing high-throughput sequencing public datasets of KC. We identified 428 DEGs and 68 DELs shared between the two datasets and uncovered functional association changes in proteasomal ubiquitin–dependent protein catabolic process genes and pathways in KC. We uncovered the top 20 hub genes in which *FBXW11*, *FBXO9*, *RCHY1*, and *CD36* were validated by qPCR. Interestingly, we predicted that triptolide, a small-molecule drug, had the potential to bind critical genes FBXW11 and FBXO9 on different amino acid residue sites by molecular docking techniques. Subsequently, we predicted and screened four core miRNAs associated with KC and validated them by qPCR. Following this, the ceRNA regulatory network was built to select the key RNAs affecting the development of KC. Our study revealed the ceRNA posttranscriptional regulation mechanism correlated with the pathogenesis of KC and provided four miRNAs as valuable biomarkers, four mRNAs as therapeutic targets, and a new idea of drug therapy for KC.

## Materials and Methods

### High-Throughput RNA-Sequencing Datasets of KC Preparation and Screening of Differentially Expressed mRNAs and lncRNAs

High-throughput RNA-sequencing data of KC were downloaded from the Gene Expression Omnibus (GEO) database (Barrett et al., 2005) (http://www.ncbi.nlm.nih.gov/geo/; the gene expression profile accession numbers, GSE77938 and GSE151631). Since the cornea as a whole contains three cell layers, we selected sequence data of the entire cornea (including epithelium, stroma, and endothelium) in order to screen key RNA targets in KC as a whole. So, we excluded the data of sequencing only the corneal epithelium. In brief, GSE77938 contained 25 KC samples and 25 control samples (Kabza et al., 2017). GSE151631 consisted of 19 KC samples and seven control samples (Shinde et al., 2020). We removed the RNAs with a mean expression value lower than 1 and a median read count equal to 0 across all samples. Compared to the control samples with KC samples, the “DESeq2” package (Love et al., 2014) in R (version 4.0.1) software was utilized to identify the differentially expressed genes (DEGs) with thresholds of |log2 fold change| > 1.5 and FDR (*adjusted p-value*) < 0.05. Then, we used the annotation file in GTF format (Homo_sapiens.GRCh38.95.chr.gtf) to identify and annotate differentially expressed long non-coding RNA (DELs) with the thresholds of |log2 fold change| > 1.5 and FDR <0.05. Eventually, the DEGs and DELs of the two datasets were executed to take the intersection and obtained the 428 intersected DEGs and 68 intersected DELs.

### Protein–Protein Interaction Network Construction and Hub-Gene Screening

The protein–protein interaction (PPI) network of intersected DEGs was constructed by using the Search Tool for the Retrieval of Interacting Genes (STRING) database version 11.0 (https://string-db.org/) (Szklarczyk et al., 2019), with a confidence score >0.7. Furthermore, the PPI network was visualized in Cytoscape (version 3.8.2) (Shannon et al., 2003) software. Subsequently, we performed module analysis of the PPI network through the Molecular Complex Detection (MCODE) (Bader and Hogue, 2003) tool of Cytoscape software to filter the top three hub-PPI networks. In addition, the top 20 hub-genes ranked by degree were calculated by cytoHubba apps (Chin et al., 2014) of Cytoscape software.

### Functional Enrichment Analysis

The clusterProfiler (version 3.18.1) (Yu et al., 2012) package of R (version 4.0.1) software and the Metascape website (https://metascape.org/) (Zhou et al., 2019) were used to perform the Gene Ontology (GO) functional enrichment analysis in the category biological processes (BP) of intersected DEGs, and Kyoto Encyclopedia of Genes and Genomes (KEGG) pathway enrichment analysis was also performed. The *p*-value < 0.05 was selected as a statistically significant term.

### Gene Set Enrichment Analysis

GSEA was conducted to discover which specific gene sets are significantly associated with each of the two different biological states from gene expression levels (Subramanian et al., 2005). We separately performed GSEA of DEGs in each dataset through the fgsea (version 1.16.0) package (Korotkevich et al., 2021) and selected *p*. adjusted <0.05 was considered as the threshold for statistical significance.

### Molecular Docking

Triptolide, which is contained in the thunder god vine, is a diterpenoid epoxide. It has been reported that corneal fibroblasts secrete large amounts of MMPs to degrade collagen in KC (Smith et al., 2006), while triptolide can inhibit collagen degradation by downregulating the production of MMPs (Lu et al., 2003). However, whether triptolide can alleviate KC by interacting with core mRNA targets has not been reported. It is possible to reveal whether proteins and small molecules have binding sites and predict the specific location and interaction force by using molecular docking analysis. The molecular structure of triptolide was obtained from the PubChem database (Wang et al., 2017) (https://pubchem.ncbi.nlm.nih.gov/). The protein structure of FBXW11 and FBXO9 proteins was obtained from the PDB database (Burley et al., 2017) (https://www.rcsb.org/) and UniProt Consortium (2021) (https://www.uniprot.org/). Then, we used AutoDock Tools (version 1.5.6) (Morris et al., 2009) software to perform molecular deletion of water and add hydrogen and convert the original pdb file to pdbqt file format. Then, we performed the AutoDock Vina program for molecular docking. The smallest affinity energy and the root mean square deviation (RMSD) ≤ 4 were considered the optimal binding phase. Finally, we showed the binding sites of the binding complexes with pyMOL (version 2.4.0) software.

### Prediction of Core miRNAs by the Top 20 Hub Intersected DEGs and Intersected DELs

To narrow down the range of miRNAs that regulate core gene expression, we used the top 20 hub genes to predict miRNAs and intersect the results with the miRNAs predicted by using intersected DELs as the final result. We predicted that miRNAs could bind to 20 hub genes through miRTarBase (Huang et al., 2020) (http://mirtarbase.mbc.nctu.edu.tw/), starBase version 2.0 (Li et al., 2014), miRDB(Chen and Wang, 2020) (http://www.mirdb.org/) and TargetScan (Agarwal et al., 2015) (http://www.targetscan.org/). Furthermore, the miRNA targets of intersected DELs were predicted using databases starBase version 2.0 (Li et al., 2014) and DIANA-LncBase version 2.0 (Paraskevopoulou et al., 2016), both of which provided experimental evidence about lncRNA–miRNA interaction. Moreover, we only selected miRNAs that both existed in two prediction results and verified the miRNAs that are abnormally expressed in KC by quantitative real-time PCR (qPCR) in additional samples. Eventually, we obtained four miRNAs (miR-4298, miR-4494, miR-4263, and miR-4257) as the final core miRNA target results after validation.

### RNA Extraction and Quantitative Real-Time PCR

KC samples were obtained from pathological keratoconus tissue excised during corneal transplantation, and normal corneas were obtained from the Eye Bank of Zhongshan Ophthalmic Center. This study was approved by the Ethical Board Committee of the Zhongshan Ophthalmic Center (No. 2021KYPJ105). Total RNA was extracted from three KC samples and three normal cornea samples using TRIzol reagent (Thermo Fisher Science) according to the manufacturer’s instructions. Reverse transcription was executed with the PrimeScript RT Master Mix kit (TAKARA, Kusatsu, Japan). The SYBR Premix Ex Taq kit (TAKARA) to perform quantitative PCR with a StepOnePlus Real-Time PCR System (Thermo Fisher Scientific). Moreover, GAPDH offered served as the internal control. The mRNA sequences of the primers are listed below: *FBXW11*-F sequence (5′-3′): GGA​ACA​TCA​TCT​GTG​ATC​GTC​TC; *FBXW11*-R sequence (5′-3′): TGG​TAA​AGC​GGT​AAT​AAA​GTC​CC. *FBXO9*-F sequence (5′-3′): CTC​AGT​GGA​TGT​TTG​AAC​TTG​CT; *FBXO*9-R sequence (5′-3′): CCT​TTG​GTA​TCT​GCC​GAT​GTT​TT. *RCHY1*-F sequence (5′-3′): TGT​GGA​ATT​TGT​AGG​ATT​GGT​CC; *RCHY1*-R sequence (5′-3′): CAA​CAC​GGG​ATG​TGT​GAA​TGT. *CD36*-F sequence (5′-3′): CTT​TGG​CTT​AAT​GAG​ACT​GGG​AC; *CD36*-R sequence (5′-3′): GCA​ACA​AAC​ATC​ACC​ACA​CCA. As for miRNA, total miRNA was isolated from three KC samples and three normal cornea samples using the miRcute miRNA Isolation Kit (DP501, TIANGEN BIOTECH, BEIJING) following the manufacturer’s protocols. Reverse transcription was performed with the miRcute Plus miRNA First-Strand cDNA Kit (KR211, TIANGEN BIOTECH, BEIJING), and quantitative PCR was performed using the miRcute Plus miRNA qPCR Kit (SYBR Green) (KR411, TIANGEN BIOTECH, BEIJING). U6 served as the internal control. The sequences of the primer used for qRT-PCR are as follows: Has-miR-4257 primer sequence from TIANGEN Primer Library (www.tiangen.com, catalog number: CD201-0476); Has-miR-4494 sequence (5–3′): CCA​GAC​UGU​GGC​UGA​CCA​GAG​G; primer sequence (5–3′): CCA​GAC​TGT​GGC​TGA​CCA​GAG; Has-miR-4263 sequence (5–3′): AUU​CUA​AGU​GCC​UUG​GCC; primer sequence (5–3′): ACG​GAT​TCT​AAG​TGC​CTT​GGC; Has-miR-4298 sequence (5–3′): CUG​GGA​CAG​GAG​GAG​GAG​GCA​G; primer sequence (5–3′): CTG​GGA​CAG​GAG​GAG​GAG​G.

### Construction of the KC-Associated lncRNA–miRNA–mRNA Network

**FIGURE 1:**
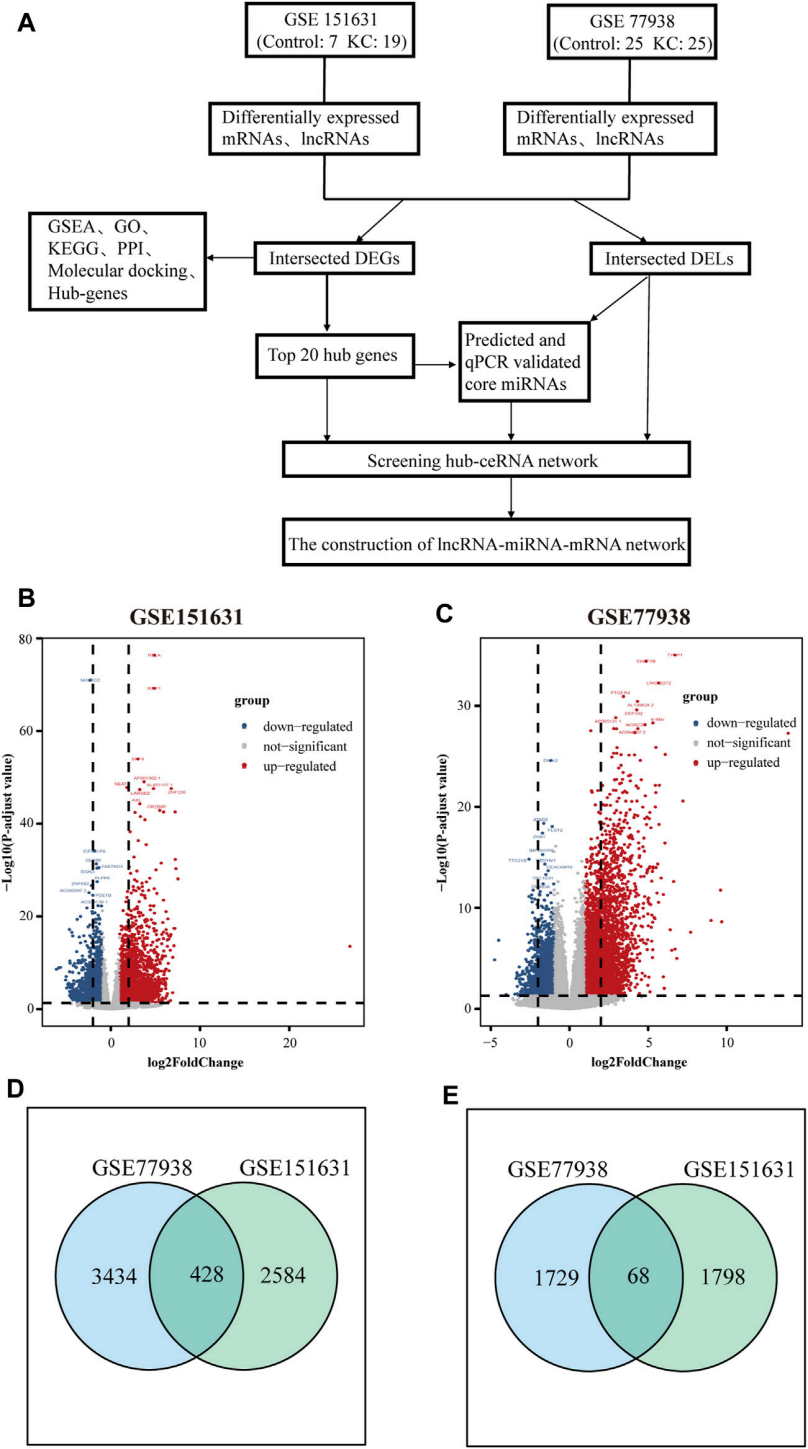
Screening workflow and results of DEGs and DELs. **(A)** Flowchart of constructing the ceRNA network. **(B)** Volcano plot showing the DEGs and DELs identified from GSE151631. **(C)** Volcano plot showing the DEGs and DELs identified from GSE77938. Note: the gray dots represent genes with no significant changes, and the blue dots and red dots represent the downregulated and upregulated genes in KC samples, respectively. **(D)** Intersection of DEGs of GSE151631 and GSE77938 datasets. **(E)** Intersection of DELs of GSE151631 and GSE77938 datasets.

After the aforementioned screening, we found that 15 of the top 20 hub genes and eight intersected DELs that bind to miRNA meet the requirements, so we constructed the hub ceRNA network using the remaining 15 hub genes and eight intersected DELs. In addition, considering that miRNAs may also bind to intersected DEGs outside the top 20 hub genes, we then used the four miRNAs to predict possible mRNA-binding targets and take intersections with our remaining intersected DEGs to obtain the overall ceRNA regulatory network through miRTarBase, starBase, miRDB, and TargetScan. The suitable intersected DEGs (expression trends were opposite to those of the four miRNAs), four validated miRNAs, and intersected eight DELs were used to construct the overall lncRNA–miRNA–mRNA (ceRNA) network, which was visualized by Cytoscape software. The flow chart (Figure 1A) delineated the whole process of ceRNA network construction.

### Statistical Analysis

RNA-seq data statistical analyses were performed using R (version 4.0.1). RT-qPCR data were reported as mean ± standard deviation (SD), and statistical significance was calculated using 2-tailed *t*-tests (SPSS Statistics Version 22.0, Armonk, NY, United States). *p* < 0.05 was considered statistically significant.

## Results

### The Screening Results of DEGs and DELs in KC

**FIGURE 2:**
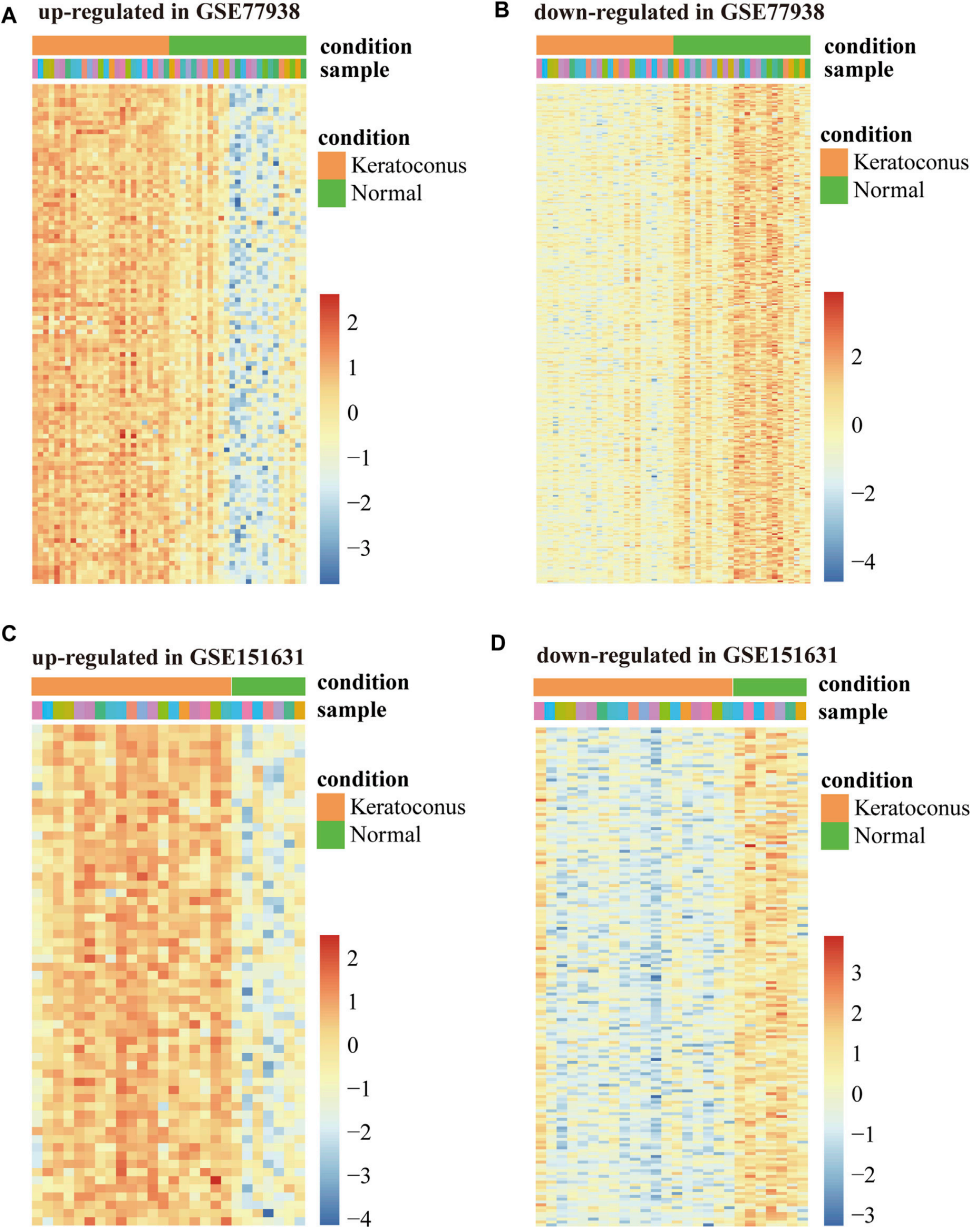
Heatmap of DEGs in GSE77938 and GSE151631. **(A)** Heatmap showing upregulated intersected DEGs in GSE77938. **(B)** Heatmap showing downregulated intersected DEGs in GSE77938. **(C)** Heatmap showing upregulated intersected DEGs in GSE151631. **(D)** Heatmap showing downregulated intersected DEGs in GSE151631.

In total, we analyzed the high-throughput RNA-sequencing data of GSE77938 and GSE151631 according to the flow chart (Figure 1A), which included a total of 44 KC samples and 32 control samples. In detail, the DEGs and DELs were separately identified in the two datasets and shown in the Volcano Plot (Figures 1B,C). Furthermore, we revealed that 428 intersected DEGs and 68 intersected DELs were shared between the two datasets through the Venn diagram (Figures 1D,E). In addition, we separately used heatmaps to reveal the expression pattern of upregulated and downregulated intersected DEGs in the two datasets (Figures 2A–D).

### GSEA of DEGs in Two Datasets

**FIGURE 3:**
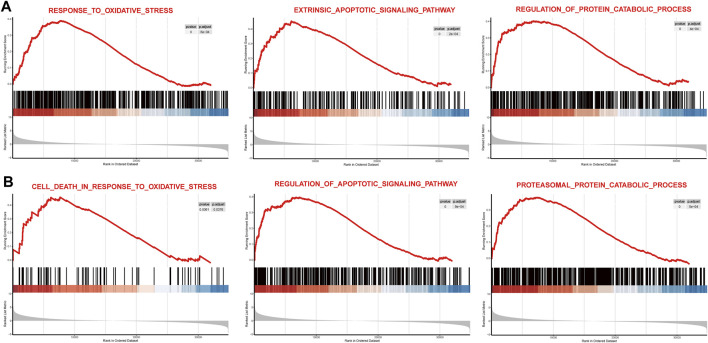
Main function gene set enrichment plots by GSEA in GSE77938 and GSE151631. **(A)** GSEA enrichment plots for representative pathways upregulated in KC compared to control in GSE77938. **(B)** GSEA enrichment plots for representative pathways upregulated in KC compared to control in GSE151631.

To observe the overall activated functions of the DEGs in a single dataset, GSEA was performed on two datasets separately. We found that the main activated functions of DEGs were involved in response to oxidative stress, extrinsic apoptotic pathway, and regulation of the protein catabolic process of GSE77938 (Figure 3A), and GSE151631 results indicated that oxidative stress, apoptotic signaling pathway, and proteasomal protein catabolic process were activated in KC samples (Figure 3B). *P-adjusted* values for all aforementioned terms were less than 0.01. These results collectively suggested that oxidative stress, cell apoptosis, and proteasomal protein catabolic process might play a vital role in the progression of KC.

### Functional Enrichment Analysis of Intersected DEGs

**FIGURE 4:**
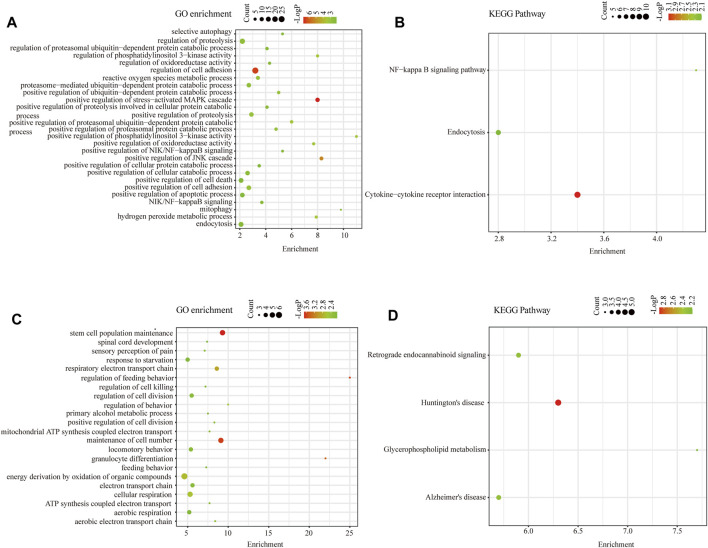
Functional enrichment analysis of intersected DEGs. **(A)** GO Biological Process enrichment analysis of upregulated intersected DEGs in KC. **(B)** KEGG pathway enrichment analysis of upregulated intersected DEGs in KC. **(C)** GO Biological Process enrichment analysis of downregulated intersected DEGs in KC. **(D)** KEGG pathway enrichment analysis of downregulated intersected DEGs in KC.

To discover the common transcription-level changes in KC and reveal the role of its functional pathways, we performed GO functional and KEGG pathway enrichment analyses of intersected DEGs (Figure 4). We found that GO functional enrichment of upregulated intersected DEGs was mainly related to oxidative stress, cell adhesion, positive regulation of proteasomal ubiquitin–dependent protein catabolic process, proteolysis, apoptotic process, PI3K signaling, and NF-kB signaling, while the KEGG enrichment pathway was mainly related to NF-kB pathway and endocytosis (Figures 4A,B). Meanwhile, GO functional enrichment of downregulated intersected DEGs was involved in ATP synthesis coupled electron transport, cellular respiration, respiratory electron transport chain, and stem cell population maintenance, while KEGG pathways were enriched in some neurodegenerative diseases such as Huntington's disease and Alzheimer's disease (Figures 4C,D). These results revealed that oxidative stress, positive regulation of proteasomal ubiquitin–dependent protein catabolic process, and cell apoptosis may play a critical role in the pathogenesis of KC. Moreover, the enriched entries for neurodegenerative diseases suggested that KC might have similar features to degenerative diseases.

### PPI Network Construction and Visualization

**FIGURE 5:**
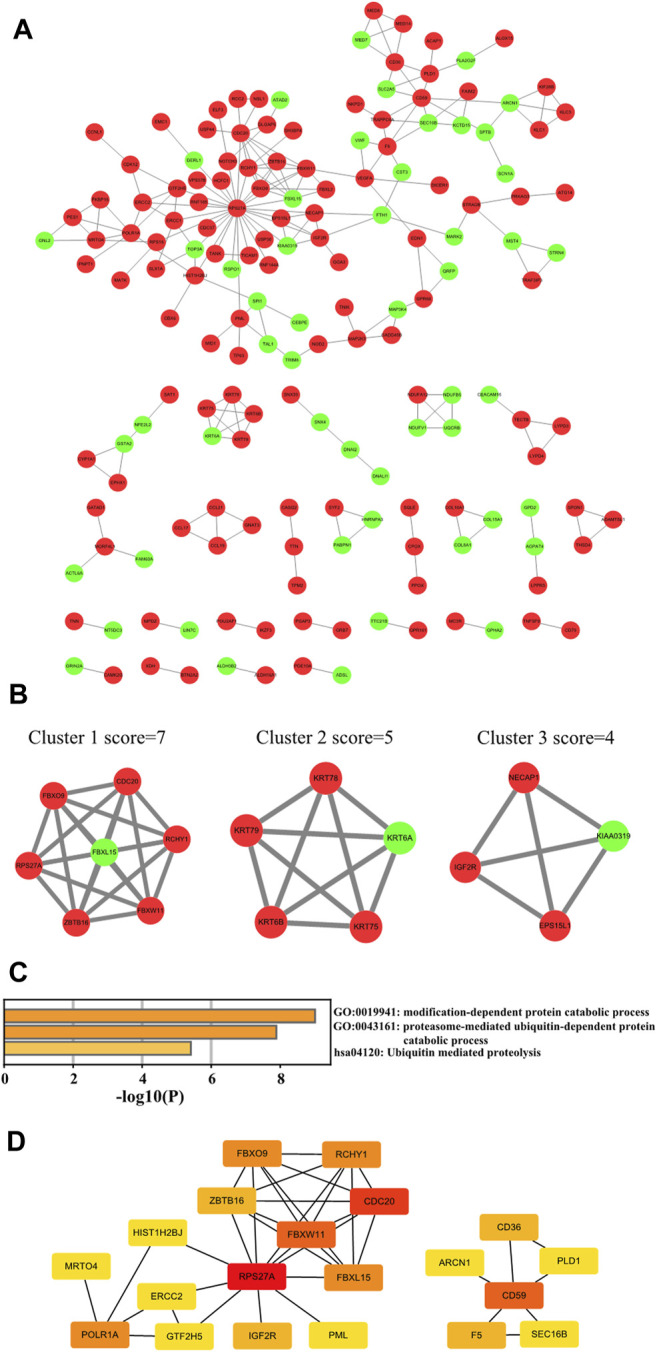
PPI network construction of intersected DEGs. **(A)** Cytoscape software visualizing the PPI network. Note: *Red* means upregulated and *light green* means downregulated. **(B)** Top three cluster networks in PPI. **(C)** Functional enrichment analysis of Cluster 1 core network genes. **(D)** Top 20 hub genes in the PPI network of intersected DEGs in KC. Note: the color change from *red* to *yellow* represents the hub gene degree score from high to low.

To figure out which hub genes play functional roles in the pathogenesis of KC, we constructed a PPI network of the intersected DEGs using the STRING online tool and visualized it by Cytoscape software (Figure 5A). The proteins that disconnected from any other protein in the PPI network were removed. Overall, we revealed 168 nodes and 221 edges in this network, which comprised 116 upregulated genes and 52 downregulated intersected DEGs. The top three sub-networks were analyzed with the MCODE algorithm (Figure 5B). The C1 cluster, which is at the core of the PPI network, contains genes *RPS27A*, *FBXO9*, *FBXW11*, *FBXW11*, *FBXL15*, *CDC20*, *RCHY1*, and *ZBTB16.* Functional enrichment of Cluster 1 genes is mainly involved in the modification-dependent protein catabolic process, proteasome-mediated ubiquitin-dependent protein catabolic process, and ubiquitin-mediated proteolysis (Figure 5C). Subsequently, the top 20 hub DEGs were filtered out with degree ≥5 through Cytoscape plugin cytoHubba (Figure 5D), which might play significant roles in the pathogenesis of KC.

### Molecular Docking Suggested that Triptolide Could Bind to FBXW11 and FBXO9 Amino Acid Residues

**FIGURE 6:**
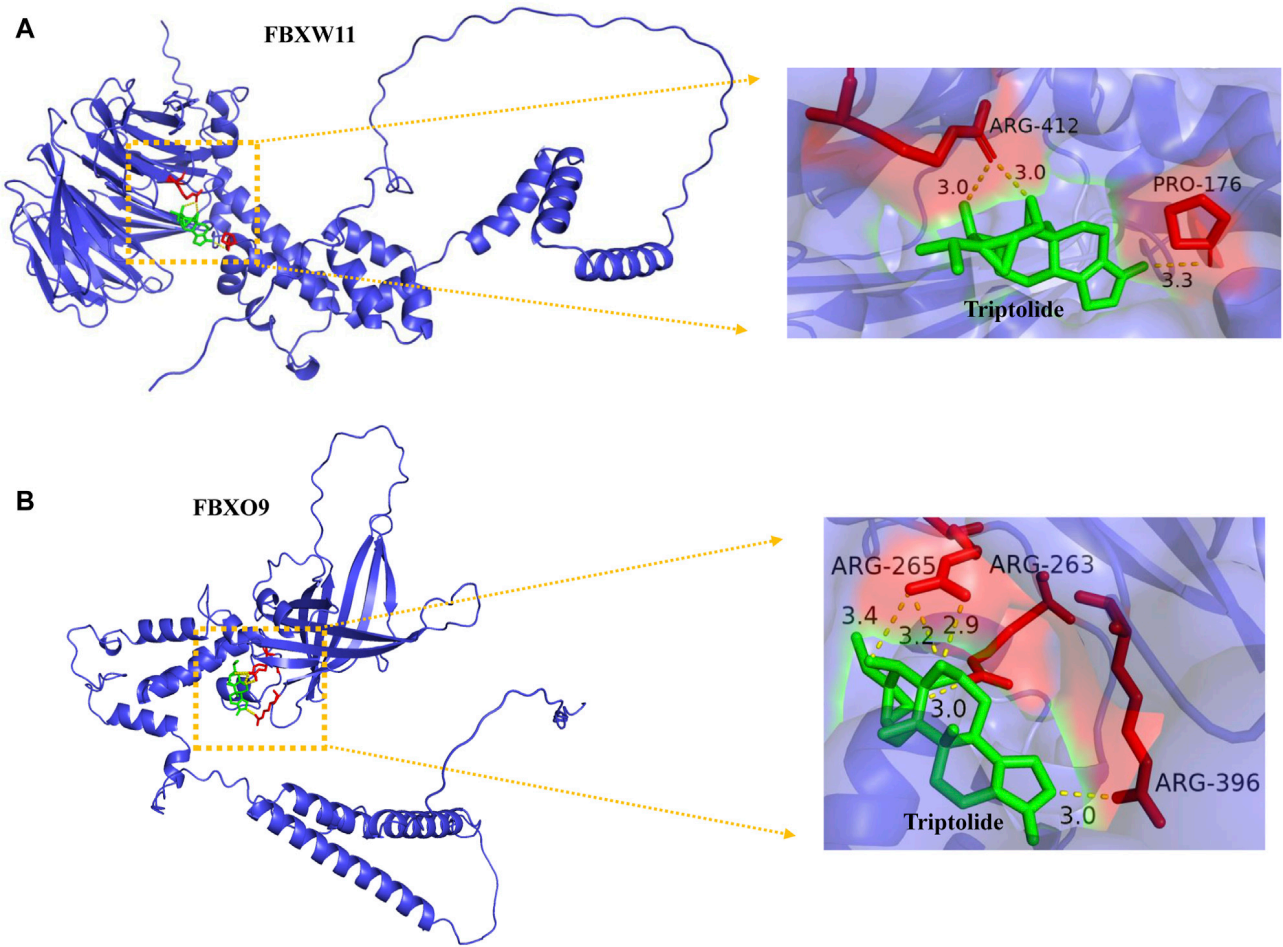
Binding of triptolide to the core target FBXW11, and FBXO9 using molecular docking analysis. **(A)** Hydrogen bond sites formed between triptolide and FBXW11 protein on ARG-412 and PRO-176. **(B)** Hydrogen bond sites formed between triptolide and FBXO9 protein on ARG-265, ARG-263, and ARG-396. *Green* represents triptolide, while *red* represents amino acid residue binding sites.

Our enrichment results revealed that the ubiquitin-dependent protein catabolic process was upregulated in KC and proteins encoded by FBXW11 and FBXO9 constitute subunits of the ubiquitin–protein ligase complex. We speculated that FBXW11 and FBXO9 which belong to the top 20 genes might play an important role in the development of KC and whether triptolide could alleviate the progression of KC by binding to these genes. Here, we revealed that triptolide could bind to FBXW11 and FBXO9 amino acid residues by molecular docking. The binding affinity energy of triptolide to each protein structure is shown in Table 1. Molecular docking analysis predicted that triptolide could interact with the FBXW11 protein on ARG-412 and PRO-172 (Figure 6A) and combine with the FBXO9 protein on ARG-265, ARG-263, and ARG-396 by forming hydrogen bond on the site (Figure 6B).

**TABLE 1:** MOE scores of FBXW11 and FBXO9 protein with triptolide.

Protein	Mode	Affinity (Kcal/mol)	Dist from Rmsb* l.b	Best Mode Rmse u.b
FBXW11	1	−8.6	0	0
	2	−8.1	2.129	3.794
	3	−7.5	2.202	7.314
	4	−7.5	1.728	6.998
	5	−7.5	3.881	5.43
	6	−7.5	56.74	58.71
	7	−7.3	54.906	58.2
	8	−7.3	31.01	32.829
	9	−6.9	56.303	59.913
FBXO9	1	−7.9	0	0
	2	−7.4	2.617	4.071
	3	−7	30.675	32.675
	4	−6.9	2.601	4.455
	5	−6.9	11.773	14.28
	6	−6.8	18.356	20.954
	7	−6.7	17.875	21.024
	8	−6.6	26.723	28.586
	9	−6.6	18.211	20.107

### Experimental Verification (qPCR) of Four Critical mRNAs and Four Core miRNAs

**FIGURE 7:**
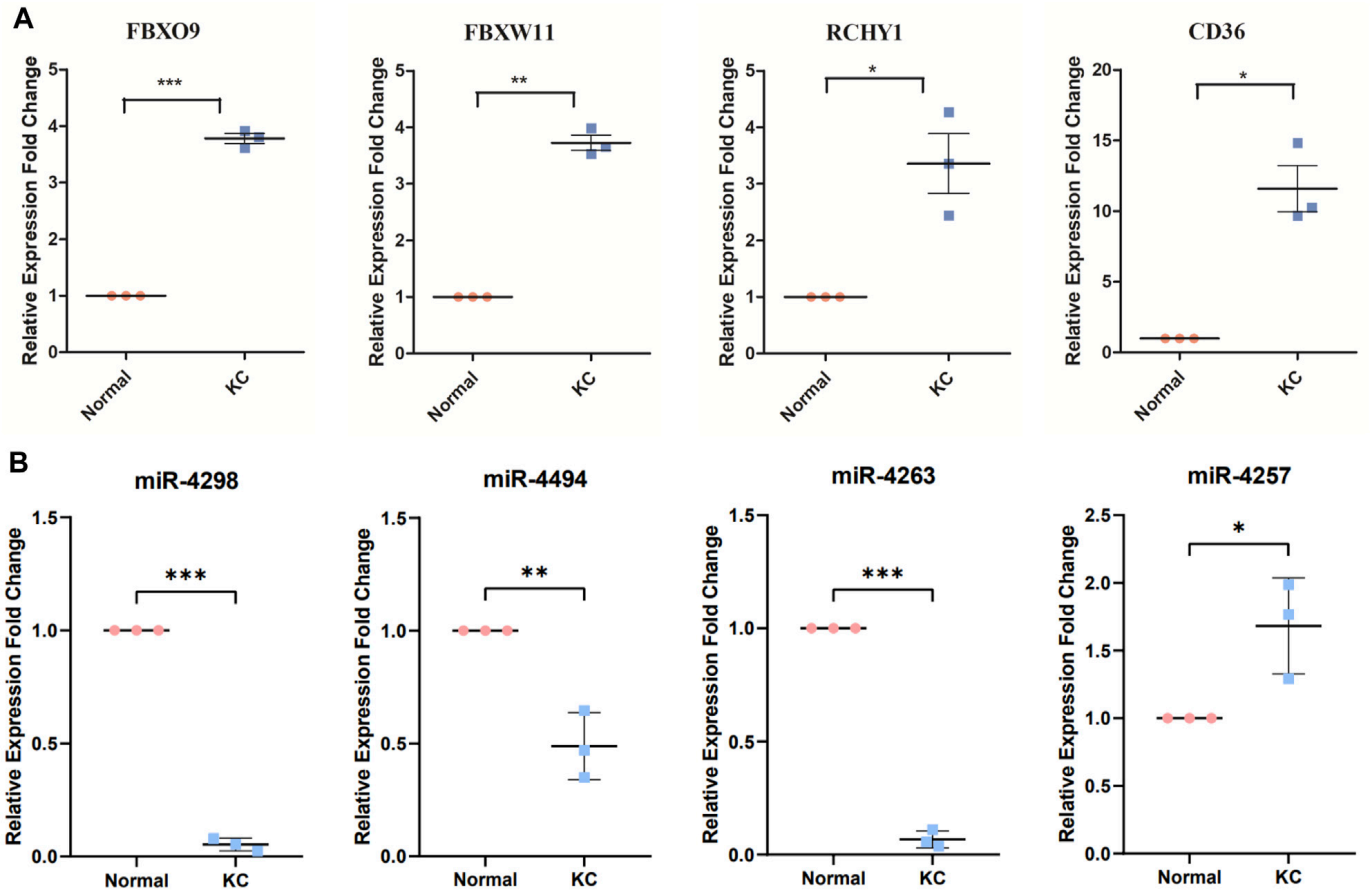
Validation of core-predicted miRNAs using qRT-PCR. **(A)** Q-PCR showed critical genes FBXO9, FBXW11, RCHY1, and CD36 mRNA upregulated in KC. **(B)** Figure showed miR-4298, miR-4494, and miR-4263 downregulated in KC, while miR-4257 was upregulated in KC. Note: *Pink* represents normal samples and *blue* represents KC samples. *p < 0.05, **p < 0.01, and ***p < 0.001.

Since enrichment analysis results suggested significant enrichment of the proteasome-mediated ubiquitin-dependent protein catabolic process, and among the top 20 genes, *FBXO9*, *FBXW11*, and *RCHY1* encode proteins that are closely related to the function of E3-dependent ubiquitination, we selected these genes for qPCR validation. The PCR results revealed that *FBXO9*, *FBXW11*, *RCHY1*, and *CD36* mRNA levels were upregulated in KC (*p* < 0.05, Figure 7A), which were consistent with our enrichment analysis results. Collectively, the expression levels of the four core miRNAs after screening (miR-4298, miR-4494, miR4263, and miR-4257) were verified by qPCR. We revealed that miR-4257 was upregulated in KC, while miR-4298, miR-4494, and miR-4263 were downregulated in KC, with statistically significant differences (*p* < 0.05, Figure 7B).

### Construction of the CeRNA Network in KC

**FIGURE 8:**
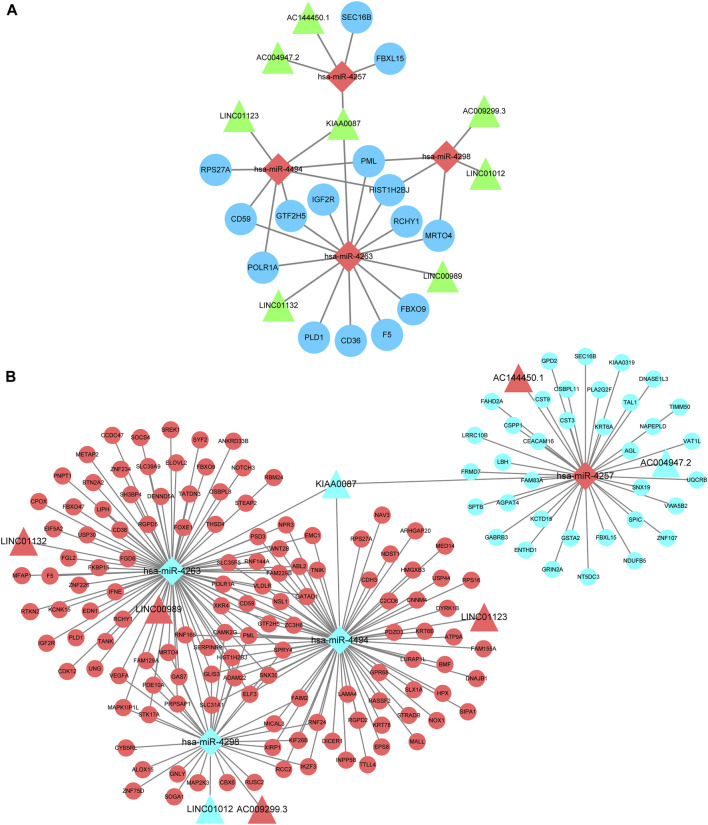
CeRNA regulatory network construction. **(A)** Hub ceRNA network which contains 15 top hub genes associated with KC. Note: *Circles* represents hub genes, *rhombus* represents intersected miRNAs, and *triangles* represent intersected lncRNAs. **(B)** Cytoscape software visualizes the overall lncRNA–miRNA–mRNA regulatory network. Note: *Circles* represent intersected mRNAs, *rhombus* represents intersected miRNAs, and *triangles* represent intersected lncRNAs. *Red* means upregulated and *light blue* means downregulated.

Eligible RNAs were used to construct a core KC-associated ceRNA regulatory network which included 15 hub genes, four validated miRNAs, and eight intersected DELs (Figure 8A). Subsequently, we constructed a global ceRNA regulatory network based on the predicted miRNA–mRNA interactive pairs of the remaining intersected DEGs that could bind to the four core miRNAs and those with expression trends opposite to the four core miRNAs. Overall, the network included 129 upregulated and 36 downregulated intersected mRNAs, five upregulated and three downregulated intersected lncRNAs, and one upregulated miRNA and three downregulated miRNAs (Figure 8B).

## Discussion

The development of high-throughput sequencing technology and bioinformatics enables us to screen for key genes and therapeutic targets of disease, providing the possibility to understand the molecular mechanism of KC in depth. In this study, by analyzing high-throughput sequencing data of multiple samples and screening hub genes, we explored critical functional changes in KC and constructed a posttranscriptional regulatory network to reveal the mechanism in KC.

According to the results of the analysis, 428 DEGs and 68 DELs were shared in the two datasets. GSEA was performed on the two datasets separately, revealing that oxidative stress, apoptotic signaling pathway, and protein catabolic process were activated in KC. GO enrichment analysis of intersected DEGs further implied that the critical transcriptional level changes in KC were concentrated in oxidative stress, positive regulation of proteolysis involved in cellular protein catabolic process, proteasome-mediated ubiquitin-dependent protein catabolic process, and apoptotic process. The KEGG pathway was involved in the NF−kappa B signaling pathway and some neurodegenerative disease, which was consistent with similarities of KC with other neurodegenerative diseases that had been reported (Chaerkady et al., 2013) using proteome analysis. A previous study has reported that levels of oxidative stress markers such as nitrites and lipid peroxidation increased, while total antioxidant capacity and glutathione levels decreased in KC, suggesting that oxidative stress might be involved in the progression of KC (Arnal et al., 2011). It has also been observed that KC cells exhibit high levels of oxidative stress in *in vitro* models (Karamichos et al., 2014). In addition to the activation of oxidative stress, other biological processes have been observed in KC. Keratocytes have morphologic changes of apoptosis which have been detected by transmission electron microscopy in KC rather than normal corneas (Kim et al., 1999). Kaldawy et al.(Kaldawy et al., 2002) uncovered the phenomenon of apoptosis through TUNEL staining and proposed apoptosis as a form of cell death in KC. These findings are consistent with the results of our enrichment analysis. Furthermore, it has been reported that levels of cathepsins B and G increased (Zhou et al., 1998) and collagen catabolic and aminoglycan catabolic processes were upregulated in KC (Hao et al., 2020). In our study, we innovatively revealed that proteasomal ubiquitin–dependent protein catabolic process was upregulated in KC, which might partially explain the previous findings that protein digesting and catabolic process are increased in KC.

Intersected DEGs were used to construct the PPI network, and the top three core networks were screened. Functional enrichment analysis revealed that Cluster 1 core network genes are mainly involved in proteasome-mediated ubiquitin-dependent protein catabolic process and ubiquitin-mediated proteolysis. It has been reported that triptolide can inhibit proteasomal activity and induce cell apoptosis in human breast and prostate cancer cell lines (Lu et al., 2011) and can also inhibit collagen degradation by downregulating the production of MMPs in corneal fibroblasts (Lu et al., 2003). In addition, in the Cluster 1 core network genes, *FBXO9* and *FBXW11*, which are related to the ubiquitin-proteasome system (UPS), were up-regulated in KC. Therefore, we attempt to explore whether triptolide could act on these core proteins. The results showed that triptolide could bind to FBXO9 and FBXW11 proteins *via* different amino acid residue sites. Thus, we proposed a hypothesis that it is possible to alleviate the symptoms and progression of KC by using certain herbal medicines to act on key mRNA targets. These findings provided new ideas for improving the drug treatment of KC.

Top 20 hub genes, including *RPS27A*, *FBXO9*, *FBXW11*, *FBXL15*, *RCHY1*, *CD59*, *CD36*, *PML*, *IGF2R*, and *SEC16B*, were screened by MCODE and cytoHubba. It has been demonstrated that RPS27A increases in response to DNA damage stress, amplifies the signals of p53 that induced cell cycle arrest (Nosrati et al., 2015), and activates p53 signaling in response to ribosomal stress (Sun et al., 2011). Moreover, RPS27A is synthesized as a fusion protein with ubiquitin (Redman and Rechsteiner, 1989), suggesting that RPS27A is involved in regulating stress stimuli and UPS. FBXO9, FBXW11, and RCHY1 belong to UPS. Ubiquitin signaling is involved in NF-kB pathway activation by degrading NF-kB inhibitors and processing precursors of NF-kB and activating IkB kinase (Chen, 2005). The disturbance of the UPS system is also related to degenerative disease (Gadhave et al., 2016). More importantly, inhibition of the ubiquitin-proteasome system downregulates matrix metalloproteinases 2 (MMP2) and MMP9 expression to affect extracellular matrix (ECM) disorder and results in rat cardiac fibroblast remodeling (Meiners et al., 2004). This implied that upregulated proteasome-mediated ubiquitin-dependent protein catabolic process might activate MMP2 and MMP9 expression, which was consistent with the upregulation of MMP2 and MMP9 in KC (Smith et al., 2006; Shetty et al., 2015). It has been reported that CD36 may serve as a biomarker for conjunctival inflammation (Soriano-Romaní et al., 2015) and a critical modulator of proinflammatory and oxidative stress in hypercholesterolemic CKD (Okamura et al., 2009). Furthermore, PML exerts pro-apoptotic function with the p53 regulatory pathway in cancer suppression and is essential for multiple stress-activated apoptosis (Guo et al., 2000; Salomoni and Pandolfi, 2002). Notably, Xuefeng et al. revealed that IGF2R exerted anti-inflammatory effects in the inflammatory phenotype of macrophages, and the expression level of IGF2R increases during corneal wound healing (Wang et al., 2020). These findings suggested that corneal damage and repair might be concurrent during KC progression.

**FIGURE 9:**
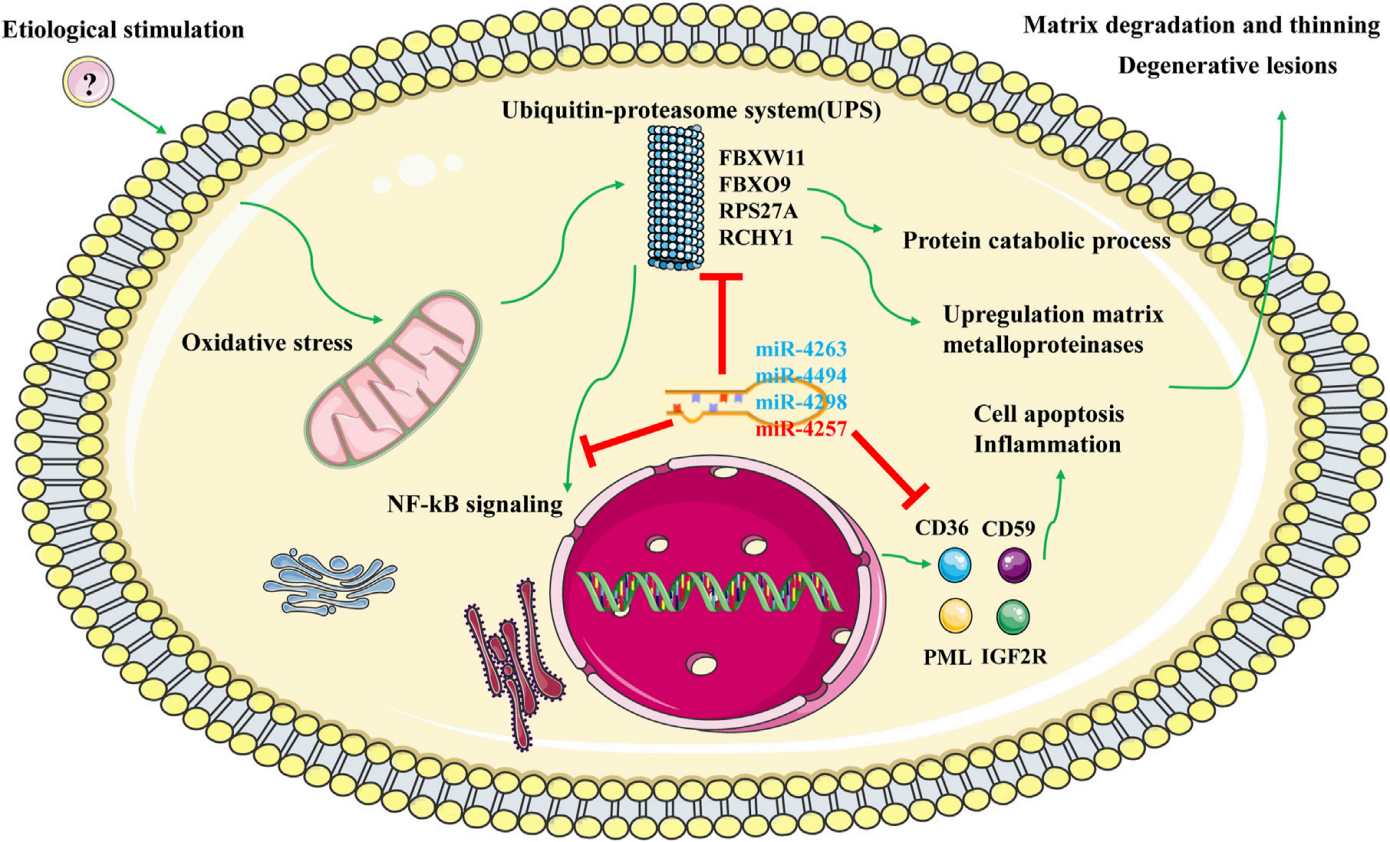
Potential regulatory mechanism of KC. Note: *Green* represents activation effects; blue represents down-regulated miRNA; *red* represents upregulated miRNA.

Based on the functional enrichment of the intersected DEGs and the functions of the aforementioned hub genes, we proposed potential pathogenesis of KC (Figure 9). When cells are affected by external etiologies, an intracellular oxidative stress response is triggered, which further activates the UPS system and promotes activation of the NF-kB signaling pathway, subsequently upregulating the protein catabolic process and increasing the expression levels of MMPs and some molecules involved in cell apoptosis and inflammation response. Furthermore, it leads to a phenotype in which KC exhibits stromal thinning and degenerative lesions. During this process, four core miRNAs might play a corresponding regulatory role. Since there are few studies on these four core miRNAs, we then discuss them in the context of the expression trends of their target genes. Under normal physiological conditions, miRNA and mRNA expression levels are normal and maintain normal cellular functions by interacting with each other. However, we confirmed that miR-4263, miR-4298, and miR-4494 were downregulated in KC, implying that the expression of the target genes they bind was upregulated. According to the ceRNA network, hub genes such as *RPS27A*, *FBXO9*, *RCHY1*, *CD59*, *CD36*, and *PML* were upregulated, leading to oxidative stress, proteasome-mediated ubiquitin-dependent protein catabolic process, and apoptotic process. In addition, we verified that miR-4257 was upregulated in KC, suggesting that its target mRNA such as SEC16B and FBXL15 were downregulated in KC. SEC16B, an endoplasmic reticulum (ER) stress–inducible gene, has a role in COPII coat dynamics (Sprangers and Rabouille, 2015). Kentaro et al.(Oh-hashi et al.(2021) revealed that the SEC16B gene responded well to ER stress–inducing stimuli, and disturbances in SEC16B expression might lead to ER stress response disorder, resulting in cellular and tissue dysfunctions. Moreover, we screened eight lncRNAs that might bind to four core miRNAs, among which LINC01132, LINC00989, AC009299.3, LINC01123, and AC144450.1 were upregulated, while LINC01012, KIAA0087, and AC004947.2 were downregulated in KC. In more detail, miR-4263 might be sponged by LINC01132 or LINC00989, miR-4298 might be bound by LINC01012 or AC009299.3, miR-4494 might bind to LINC01123 or KIAA0087, and miR-4257 might interact with AC144450.1 or AC004947.2, which in turn affects the expression of mRNA. Overall, after hierarchical screening and validation and a combination of functional enrichment analysis and interaction relationship, we revealed a hub ceRNA regulatory network which included the top 15 hub genes, four core miRNAs, and eight lncRNAs.

There are also some limitations to our study. Although we obtained the critical RNAs to construct the ceRNA regulatory network after hierarchical screening, the lncRNA–miRNA–mRNA interaction relationship among them still needs further experimental validation. In addition, the mechanisms of how triptolide acts on the corresponding targets and alleviates KC still need further investigation.

In conclusion, we revealed crucial biological processes, especially oxidative stress and proteasome-mediated ubiquitin-dependent protein catabolic process involved in the pathogenesis of KC. In addition, we uncovered that miR-4257, miR-4494, miR-4263, and miR-4298 could serve as KC biomarkers, while *FBXW11*, *FBXO9*, *RCHY1*, and *CD36* could serve as therapeutic targets. Furthermore, we constructed a complete and detailed ceRNA regulatory network of KC based on large samples, which is a valuable resource for screening critical RNAs that play functional roles in the pathogenesis of KC. Our study enriches the posttranscriptional regulation mechanism of non-coding RNAs in KC and provides core RNA therapeutic targets and new ideas for drug treatment of KC.

## Data Availability

The original contributions presented in the study are included in the article/Supplementary Material, further inquiries can be directed to the corresponding author.
